# Supplementary material for: Regulation of adenylyl cyclase 5 in striatal neurons confers the ability to detect coincident neuromodulatory signals
Source: PLoS Comput Biol. 2019 Oct 30;15(10):e1007382. doi: 10.1371/journal.pcbi.1007382 (PMC6821081; doi:10.1371/journal.pcbi.1007382)
Supplement: S1 Text — (PDF) [file pcbi.1007382.s018.pdf]

The allosteric exclusion scheme inherently lacks the ability for coincidence detection

To show this we make the following simplifications:

1. During a Da ↑ and a ACh ↓, respectively, the rise in [$G\alpha_{olf}$] and drop in [$G\alpha_{i}$] are assumed to be square-shaped, i.e. the interactions between the G proteins and AC5 are assumed to be sufficiently fast so that the transient steady state levels in the network are achieved quickly and last for the whole duration of the signals
2. During the Da ↑, enough $G\alpha_{olf}$ is produced to occupy all AC5, i.e. [$G\alpha_{olf}$] >> [$G\alpha_{i}$] and [$G\alpha_{olf}$] is at a saturating level.
3. During the ACh ↓, [$G\alpha_{i}$] drops to approximately 0, i.e. all available AC5 is disinhibited from $G\alpha_{i}$;

We consider what happens in the limiting cases of 'perfect' stimulation and inhibition of AC5, i.e. $\alpha_{\mathrm{G_{olf}}} \to \infty$ and $\alpha_{\mathrm{G_{i}}} = 0$. With these assumptions only the effect of the structure of the regulatory scheme on coincidence detection is isolated.

We now determine the synergy of this scheme. The expression for the synergy is repeated here for convenience:

$$S(t) = \frac{k_c(Da\uparrow, ACh\downarrow, t)}{k_c(Da\uparrow, t) + k_c(ACh\downarrow, t) - k_{c,ss}}$$

Each of the terms in the expression for the synergy are as follows. For Da ↑ + ACh ↓, there is no $G\alpha_{i}$ in the system during a ACh ↓, so all AC5 is occupied by the produced saturating concentration of $G\alpha_{olf}$.

$$k_c(Da\uparrow + ACh\downarrow, t) = \; k_{c,\ AC5 \cdot G\alpha \mathrm{olf}} = \alpha_{G\mathrm{olf}}\; k_{c,\ AC5}$$

For Da ↑ alone, there is enough $G\alpha_{olf}$ to outcompete $G\alpha_{i}$ in the occupation of AC5, and AC5 is saturated with $G\alpha_{olf}$, which yields the same result as for k_c(Da↑ + ACh↓):

$$k_c(Da\uparrow, t) = \; k_{c,\ AC5 \cdot G\alpha \mathrm{olf}} = \alpha_{G\mathrm{olf}}\; k_{c,\ AC5}$$

For ACh ↓ alone, there is no $G\alpha_{i}$ in the system and AC5 is partly occupied by any resting-state levels of $G\alpha_{olf}$:

$$k_c(ACh\downarrow, t) = p_4\; k_{c,AC5} \; + \; p_5 k_{c,AC5 \cdot G\alpha_{\mathrm{olf}}},$$

where

$$p_4 = \frac{[AC5{\cdot}G\alpha_{\mathrm{olf}}]_{\mathrm{ACh}\downarrow}}{[AC5]_{\mathrm{ACh}\downarrow}+[AC5{\cdot}G\alpha_{\mathrm{olf}}]_{\mathrm{ACh}\downarrow}}, \text{and}$$

$$p_5 = \frac{[AC5{\cdot}G\alpha_i]_{\mathrm{ACh}\downarrow}}{[AC5]_{\mathrm{ACh}\downarrow}+[AC5{\cdot}G\alpha_i]_{\mathrm{ACh}\downarrow}} = 1 - p_4.$$

The steady state value for k_c is given under the section 'Average catalytic rate' in the Methods of the main text. Substituting these expressions in the expression for the synergy, and taking the limit $\alpha_{\mathrm{G_{olf}}} \to \infty$ yields:

$$S_{\alpha_{\mathrm{Golf}} \to \infty} \to \frac{1 - p_2}{1 - p_2 + p_5 - p_2} < 1.$$

The synergy is always less than 1 since $p_5 > p_2$, i.e. p_5 is the proportion of AC5 $\cdot$ $G\alpha_{olf}$ when there is no $G\alpha_i$ in the system, which is always greater than p_2, the proportion of AC5 $\cdot$ $G\alpha_{olf}$ in the resting state when there is G_i in the system.

An example of this result is given in Fig. S16, where the concentration of G_{olf} has been made very high to mimic the conditions used for the mathematical derivation. The duration of the signals is also very long to make the (transient) steady state levels evident. As shown in Fig. S16A and S16B, in the case of a Da $\uparrow$ the high amount of $G\alpha_{olf}$ indeed outcompetes almost all of $G\alpha_i$ in occupying the available AC5, and this input produces a similar average k_c as the case of Da $\uparrow$ + ACh $\downarrow$. The synergy in this scenario is less than 1 (Fig. S16F). However, the allosteric exclusion scheme can be used for coincidence detection with suitable choices in the amounts of the G proteins (given a set of rate constants for binding and unbinding). This is shown in Fig. 3B of the main text and its corresponding Fig. S9, where the amount of G_{olf} is not enough to occupy all available AC5.
